# Midwives’ perceptions of communication at antenatal care using a bilingual digital dialog support tool– a qualitative study

**DOI:** 10.1186/s12884-025-07368-8

**Published:** 2025-03-13

**Authors:** Dima Bitar, Marie Oscarsson, Emina Hadziabdic

**Affiliations:** 1 Department of Health and Caring Sciences, Linnaeus University, Växjö, 351 95 Sweden; 2 Region Kronoberg, Strandvägen 8, Växjö, 351 85 Sweden; 3 Department of Health and Caring Sciences, Linnaeus University, 391 82 Kalmar, Sweden; 4 Department of Health and Caring Sciences, Faculty of Health- and Life Sciences, Linnaeus University, 351 95 Växjö, Sweden

**Keywords:** Antenatal care, Communication, Dialog support tool, Digital intervention, Migration

## Abstract

**Background:**

Sweden has a large population of migrant women, which contributes to communication challenges and, consequently, suboptimal maternity care. Compared with native-born women, migrant women have an increased prevalence of adverse pregnancy outcomes. Miscommunication and language barriers are among the reasons for these results. Thus, language barriers can also lead to providing less information to migrant women. A digital Swedish-Arabic dialog support tool was developed and tested at antenatal care, to facilitate communication between midwives and Arabic-speaking women. This study aimed to describe midwives’ perceptions of communication via Swedish-Arabic dialog support (Sadima) in antenatal care.

**Methods:**

A qualitative study was conducted with 14 midwives in antenatal care with experience communicating using a Swedish-Arabic dialog support tool. The data were collected via semi structured individual interviews and were analyzed via phenomenographic analysis.

**Results:**

The analysis resulted in three categories: (1) Dialog support - the skill of constructing bridges, comprised the main finding that dialog support facilitated communication by providing a multimodal way of communication including intercultural evidence-based content;(2) Dialog support - challengingly implementing adaptive efficiency, represented the implementation of dialog support to be time-consuming and, eventually, time-efficient when midwives gained digital skills; and (3) Women and their partners - the ability to be empowered, included the main finding of increased women’s empowerment and control over their lives by being less dependent on interpreters.

**Conclusions:**

The findings contribute to the understanding of communication via dialog support based on midwives’ experiences. This study highlights that communication via dialog support facilitates communication between midwives and Arabic-speaking women and enhances midwives’ working conditions. Within our increasingly heterogeneous societies, health care could provide support for communication via digital dialog support that is women-centered and culturally sensitive to avoid misunderstandings and delayed or incorrect treatment of migrant women.

**Supplementary Information:**

The online version contains supplementary material available at 10.1186/s12884-025-07368-8.

## Background

The number of international migrants has increased over the past five decades. The proportion of migrants is approximately 281 million [1], 128 million more than that in 1990 and more than three times greater than that in 1970 [2]. As a result of international migration, almost 20% of the population of Sweden are migrants [3]. Most of them originated from Syria, India, Poland, and Pakistan [4]. Midwives working in antenatal care (ANC) face an increasing proportion of migrant women, which creates communication challenges [5, 6]. Communication is essential for providing good healthcare and includes linguistic and cultural aspects [7]. Language proficiency is important for migrant integration in health, education, and employment and for accelerating progress toward self-sufficiency [8]. Communication and linguistic difficulties have been identified as obstacles to providing equal maternity care among migrant women [9, 10]. In addition, migrant women are more likely to have an increased prevalence of adverse pregnancy outcomes than native-born women. Miscommunication, language barriers and delays in care-seeking are the main reasons for these results [5, 6, 11, 12]. Thus, language barriers can also lead to midwives providing less information to migrant women [9, 13].

A health report by the World Health Organization (WHO) revealed that interventions in cultural mediation, using interpreters, translating information, and training and guidance for health care professionals were effective strategies for reducing communication barriers [14]. Interventions to overcome language barriers could be linguistically or culturally adapted. Linguistically adapted interventions consisted of providing written or oral information in the migrant women´s native language. Culturally adapted interventions consisted of adapting routine care to meet the cultural context of migrant women, for example, lifestyle behaviors, cultural roles and beliefs, and food preferences [15].

The literature review found previous studies concerning different linguistically or culturally adapted interventions to overcome language barriers in maternal health care (MHC) [15–21]. The interventions consisted of professional interpreters, linguistically translated brochures [15, 22], bilingual trained health care professionals [15], touch pads with pictures and text, translation applications (apps) [22–24], and pregnancy apps for linguistically diverse women [25, 26]. Another intervention was group antenatal care (gANC) for Somali-born women, which was an integrated routine ANC with language-supported birth preparation and parental education provided by an interpreter [19]. A community-based bilingual doula is another intervention to provide language assistance and support during labor and provides empowerment that complements the role of midwives [20]. The MAMAACT intervention in Denmark included educational materials and a smartphone app on warning signs of pregnancy for all women and training in intercultural communication for midwives [16]. Further intervention is a clinical model for addressing barriers to accessing MHC among migrant women. This model is performed by providing a simple connected unit of care and education to migrant women provided by a clinical health advisory with the same background as the women [18]. These interventions have proven to be feasible and acceptable [21, 27], improve migrant women’s well-being [21, 28], and provide women-centered care [29]. Furthermore, linguistically adapted interventions results in increased rates of preventive reproductive health activities, mammography, condom use [15], and cervical cancer screening [15, 17].

Owing to identified challenges in communication with migrant women [6, 9, 11, 12] and the increased migration of Arabic-speaking women (ASW) in Sweden [4], a Swedish-Arabic dialog support tool named Sadima was developed by the research team to facilitate communication between midwives and ASW at ANC (the Sadima intervention is further described in the Methods section). Despite the increase of communication interventions, a limited number of E-health apps have undergone rigorous scientific evaluation to ascertain the accuracy of the provided information and its alignment with established obstetric guidelines [25, 30]. The contribution of Sadima is the combination of being evidence-based, multimodal (linguistic/written, aural and visual/images) and culturally adjusted for migrant women. The previous study, which focused on ASW´s communication experiences via dialog support, revealed that the content was educational and reliable and provided women with a sense of security. It was time-effective and allowed opportunity for dialog, but an interpreter was needed in cases of low proficiency in Swedish [31]. However, no previous investigations have focused on midwives’ perceptions of communication via Swedish-Arabic dialog support at ANC. This study, together with a previous study [31] from the women´s perspective, can help us adopt a holistic picture of the perceptions of communication via dialog support at ANC. Therefore, this study aims to explore midwives’ perceptions of communication via Swedish-Arabic dialog support (Sadima) in antenatal care.

## Methods

### Design

A descriptive design using phenomenographic analysis by Sjöström and Dahlgren (2002) was chosen to describe midwives’ perceptions of communication using Sadima at ANC. Phenomenography investigates different ways in which various phenomena in the world are experienced, conceptualized, understood and perceived [32, 33].

### The dialog support (Sadima) intervention

The Sadima intervention was initially a research project developed by a research team at a university in Southeast Sweden. A codesign methodology was used, entailing collaboration between end-users (midwives working at ANC), researchers, and software developers. The content was culturally sensitive and norm-critical concerning gender, sexuality, and ethnicity. Sadima was developed in Swedish and Arabic. The reason for developing content in Arabic was that most migrants in Sweden were Arabic-speaking [4].

**Fig. 1:**
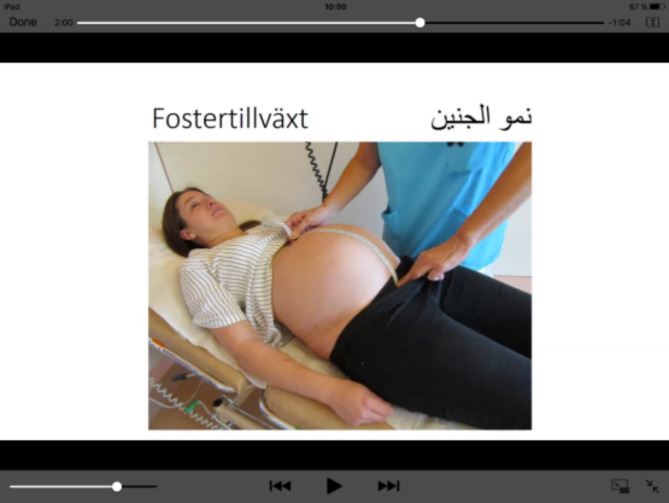
The Sadima website on the Arabic version of the information video (open access, https://sadima.lnu.se)

Sadima comprises routine information and questions used during the basic antenatal program. All the information is evidence-based, according to the National Board of Health and Welfare. Sadima contains 28 short information videos on different subjects such as introduction to Swedish MHC system, childbirth, examinations during pregnancy, breastfeeding, analgesic methods, contraception, lifestyle habits, etc. (see example in Fig. 1). Sadima also contains 98 questions including women´s backgrounds and medical- and obstetric history. The questions are followed by different answer options. Sadima is available on the website https://sadima.lnu.se. More information about Sadima was described in a previous study [34].

### Participants and procedure

A purposive sampling procedure was used in this study. The inclusion criterion was midwives working at ANC with at least two experiences using dialog support. Midwives with varying levels of experience working with migrant women were included. An invitation was sent to the heads of four ANC departments in southern Sweden. The invitation was passed to all midwives (*N* = 82) in 14 ANC clinics related to the four departments via e-mail. Study information was also provided by the first author (DB) to midwives during workplace meetings. Those who consented to participate notified the first author via e-mail. The ANC clinics were located in four different mid-sized counties. The proportion of the population in those counties is approximately 150 000-250 000 inhabitants, 15–21% of which are migrant women [3].

Fourteen midwives were recruited from seven of the 14 ANC clinics. Thirteen of the midwives were born in Sweden and one was born in England, aged 33–64. The average professional experience of ANC was two to 20 years, with a median of seven years (M = 7) (Table 1). The midwives’ communication experiences via dialog support varied from three to approximately one hundred times. The most frequent part used for dialog support was information videos. Half of the midwives used dialog support more frequently without interpreters and the other half used it more frequently as a complement to interpreters.

**Table 1:** Participating midwives

Number of participating midwives	Experience of ANC (years)	Age (years)
Midwife 1	7	49
Midwife 2	4	33
Midwife 3	20	64
Midwife 4	7	42
Midwife 5	16	52
Midwife 6	5	36
Midwife 7	2	38
Midwife 8	3	46
Midwife 9	2	37
Midwife 10	7	44
Midwife 11	9	46
Midwife 12	8	42
Midwife 13	7	35
Midwife 14	10	48

### Data collection

Data were collected between March 2022 and June 2024 via semi structured individual interviews. In developing the interview guide, the findings from the previous qualitative study [31] were used. The interview guide underwent a pilot test conducted by two midwives as well as by our expert advisory council of researchers with experience in sexual and reproductive health, qualitative research, and migration studies, which resulted in minor adjustments to the wording [35].

The interview guide contained questions such as “Tell me how it was to use the dialog support?”, “How did the dialog support affect communication with women and their partners?” and “Which pros and cons can you describe when communicating via the dialog support?”. A subsequent dialog proceeded according to the answers obtained to deepen, further develop or clarify the answers. The follow-up questions were “What do you mean by that?”, “Can you explain that further?” and “What do you think about that?”.

The interviews were conducted either at the midwives’ workplace (*n* = 9) or by digital meetings (*n* = 5) for practical reasons and midwives’ requests. The interviews lasted 24–56 min and were conducted by the first author (DB). The data collection was continued until no new data emerged or provided additional insights into the phenomena [32].

### Data analysis

The interviews were audio recorded and subsequently transcribed verbatim by the first author. Data were analyzed via phenomenographic analysis to illustrate the different ways phenomena are perceived and the logical relationships among them [32].

The analysis process comprised seven steps. The first step was *familiarization* with the data by reading the transcripts and correcting possible errors. The second step was *compilation*, where the researchers identified the most significant elements in the answers given by each midwife to a specific question. Statements were marked with a highlighter in this step. The third step was to *condense* longer statements in the interviews to find the core of each answer. The authors continued by *grouping* similar answers in a preliminary way, which was the fourth step. The fifth step involved *comparing* categories by selecting statements to identify variations and revise the initial groups. The sixth step of the analysis process involved the authors *naming* the categories to capture their essence (see example in Table 2). The seventh and last step was to *compare* the categories to find the unique character of every category, which resulted in the ways of understanding. The description categories constitute the research outcome, which shows the different ways a specific phenomenon is experienced and the logical relationship between the phenomenon [36]. Together, the descriptive categories provided an understanding of midwives’ experiences communicating with migrant women using dialog support.

Credibility in phenomenographic research relates to the relationship between the data and the categories for describing ways of experiencing a specific phenomenon [32]. The study ensured credibility by supporting the categories with quotations. All the authors took part in the analysis process. The first author identified the categories by translating and analyzing the data. The coauthors double-checked the content of the categories to confirm their relevance. Categories were discussed and adjusted until a consensus was reached and subsequently grouped into three categories [32]. The authors have different research backgrounds, but all have backgrounds in migrant women’s health. Furthermore, credibility is ensured by describing each part of the study process, which makes it possible to reproduce the study [32].

**Table 2:** Example from the process of analysis

Interview quotes	Condensation	Perceptions	Subcategories	Categories
*“…It was truly the only chance to gain time*,* because when you use an interpreter you have to say everything and you have to be on your toes at the same time to read the woman’s body language as well*,* so that you can give so little… so one small short sentence at a time for the interpreter to translate*,* while there is more flow with the little information video and then… I thought that was very good.”*	It was the only chance to gain time, because when you use an interpreter you have to say everything and you have to be on your toes at the same time to read the woman’s body language, so that you can give so little short sentence at a time for the interpreter to translate, while there is more flow with the little information video and I thought that was very good	Providing information via the dialog support is time-effective and the conversation flows better compared to communication solely via an interpreter	Time-effective way of communicating compared to use of an interpreter	Dialog support– Challengingly implementing adaptive efficiency

## Results

**Fig. 2:**
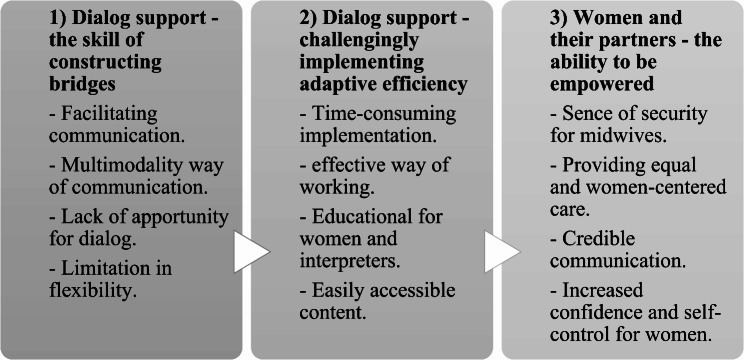
The outcome space. Three categories consisted of the varying perceptions experienced by midwives

Three categories emerged from the data analysis: (1) dialog support - the skill of constructing bridges; (2) dialog support - challengingly implementing adaptive efficiency; and (3) women and their partners - the ability to be empowered. The categories are horizontal, and no hierarchical relationships were found (Fig. 2).

### Dialog support - the skill of constructing bridges

The midwives perceived that dialog support facilitated communication and was useful for overcoming language barriers. The content followed the national basic program of ANC and facilitated the exchange of information on medical and obstetric anamnesis. An advantage was that the information was culturally adjusted for ASW including information about the MHC system, since newly arrived migrant women were described as having greater difficulties navigating Swedish MHC.

*“… I still think it* (the dialog support) *is very clear or easy to survey and as I said*,* easy to use and easy to show and not…. for some… Now it is hard to compare this one* (the dialog support) *with anything else*,* but I can experience sometimes when you show like 1177* (1177 is a website for information and services in health and care in Sweden) *to the patient or so that it is quite messy*,* that it says a lot like they… is not relevant or that may not be interesting and it takes a lot of the patient’s attention as well.”* (Midwife 4).

Further advantage expressed was that dialog support being multimodal and presented in informal language that facilitated interaction and communication with the ASW. The multimodality presentation made it possible to communicate with the ASW with various literacy levels. The images accompanying the information videos reinforced the text and speech and made it easier to understand information, especially for ASW with low language proficiency. Compared with the information in the dialog support, existing information in other languages was reported to be available mainly in written form.

*“Yes*,* I actually think that reading comprehension is probably the biggest advantage of Sadima*,* that you can hear the question. There is a lot of information translated in written form*,* then it is not certain that you can read*,* that you have a reading comprehension so that you can both read text and interpret the text and its meaning*,* so just to hear the question… that is a strong advantage of Sadima.”* (Midwife 8).

*“… If you…do not know Swedish at all or anything… eh simple things about how the body works*,* it is unfortunately the case that not everyone can that either… and then it can be truly difficult to give advanced information but eh so briefly simple*,* it always works… it would be somehow to show… for example*,* about pain relief options and a little bit of how to… what it looks like at the delivery ward”.* (Midwife 7)

The midwives perceived that communication through dialog support facilitated encounters with women during unplanned visits or when interpreters were unavailable. In these cases, it was difficult to obtain quick access to an interpreter when requested, and there was limited access to other forms of communication support.

*“And then of course the patient who came unannounced*,* of course it was… for her*,* it was certainly a great security to know that they* (midwives) *understand what I want and I know that I will be referred to the right authority and we understand each other… that we might not normally have done and then you would have had to book a phone appointment a bit urgently and it is perhaps slightly more welcoming that the patient feels that we are prepared to be able to receive a patient who comes unannounced.”* (Midwife 8).

The midwives revealed that combining dialog support with interpreters depended on the women’s language proficiency level. When encountering women with high language proficiency, the midwives perceived that the dialog support facilitated the conveyance of information and could be a foundation for further communication without an interpreter. However, when women with low language proficiency were encountered, a lack of opportunity for dialog and poorer consistency in conversation was identified and interpreter assistance was needed.

*“… It will be the correct information*,* yes*,* exactly*,* but then it might be a little worse dialog… that they watch the video and then it becomes difficult and… for them to ask questions*,* it might be easier if you tell them something yourself and they interrupt and ask how was it about that and so*,* but… they could do that*,* that they interrupt the video and ask as well*,* or something like that*,* that you say it*,* turn off the video if there’s something you’re wondering about*,* and we can talk about it.”* (Midwife 5).

Some midwives perceived a limitation in flexibility with the dialog support because its content was standardized which contributed to their inability to tailor information to individual women and specific circumstances. Instead, they were limited in providing the same information to all women, although they could limit the information by showing relevant parts of the information videos.

*“…Of course*,* when I give the information myself*,* I take into consideration is it the first child*,* is it the second child*,* the third child*,* what previous experiences do we have*,* of other births*,* and then you proceed a lot from that start point*,* that yes*,* but then I know maybe that last time*,* then we have talked about how it was…. well*,* then the information is based on that… here*,* it is the same for everyone*,* and that is good in many ways*,* but when I give the information myself*,* it becomes more nuanced or so.” (Midwife 10)*.

Furthermore, a disadvantage of providing information via dialog support was that it interrupted the interaction between the midwife and the woman, which resulted in the midwife losing track. This is in contrast to communication through interpreters, where conversations proceed continuously. However, an advantage of the interruption was that the midwives could observe the women’s interaction during the ongoing information video.

*“….I have watched the video in Swedish*,* so I know what’s being told and so*,* then I can maybe feel sometimes….it is very good*,* but sometimes it is nice to give the information yourself*,* it is like in the relationship or so*,* but….so sometimes I can miss to give the information myself and tell….it becomes like a little break in all of this….then it is not always a disadvantage that they sit and watch and you can see that it raises some thoughts sometimes*,* and some questions… It has not been a disadvantage*,* maybe it might take a few minutes before you’re in the conversation again.”* (Midwife 10).

### Dialog support - challengingly implementing adaptive efficiency

The majority of the midwives had some resistance to the implementation of dialog support; however, it was perceived as effective after gaining digital skills. The implementation was perceived as being time-consuming; thus, implementing dialog support within the designated timeframe was unfeasible. Another obstacle in implementing dialog support was that the midwives were satisfied with the available interpreter services.

*“… Because if you have plenty of time and if you can sit down before a visit and scroll through and take notes*,* that video is there and that information is there*,* then there is no problem*,* but when you have a patient and it has to be done quickly*,* you search and click around*,* oh where is the information*,* and then you cannot find it*,* then you skip it* (the dialog support) *maybe…”* (Midwife 1).

However, women’s positive attitudes toward communication with dialog support eventually impacted the extent to which the midwives used it. They felt more motivated to use dialog support in communication.

*“…There are so many times you feel inadequate*,* you feel like yes*,* but here you are and I have a mission….to help this woman in some way and then I can only reach halfway*,* because we cannot communicate. Yes*,* I have my interpreter*,* but I do not truly know*,* and then it feels so good for me with it* (the dialog support), *because then she has understood… when you see that yes*,* it turned out well… she understood me*,* she seemed satisfied with it* (the dialog support) *…”* (Midwife 4).

Sufficient time was reported to be very important for communicating with migrant women because of language difficulties. The midwives highlighted the need for additional time to address communication issues effectively and incorporate ANC check-ups. Combining dialog support with interpreters was perceived as an effective way of working since interpreting services were used for dialog instead of providing information. Furthermore, communication via dialog support resulted in fewer interpreter requirements, which led to reduced administration.

*“…It was truly the only chance to gain time*,* because when you use an interpreter you have to say everything and you have to be on your toes all the time to read the woman’s body language as well*,* so that you can give so little… so one small short sentence at a time for the interpreter to translate*,* while there is more flow with the little information video and then… I thought that was very good.”* (Midwife 3).

Providing information via dialog support created a learning opportunity for the interpreters, which was perceived to facilitate the work of both the interpreters and midwives. When interpreters lack insufficiencies in their medical terminology, midwives must allocate time toward elucidating the subject matter to them, which is time-consuming and inefficient.

*“… However*,* I think it should make their* (interpreters’) *work easier too… that we listen to it together and that I ask*,* do you have any further questions? eh and then the patient has*,* eh the opportunity to answer and then the interpreter also knows a little*,* what is this about*,* like that*,* because it sometimes happens that the interpreter says I do not understand*,* can you explain to me or ask me to repeat several times because they have not truly understood…”* (Midwife 1).

In addition, providing information via dialog support was also perceived as a learning opportunity for ASW. After the information video ended, the information was further discussed with women or interpreters, which enhanced communication. Some midwives expressed that improved communication could reduce emergency and unnecessary health care visits, which eventually could benefit the entire continuum of MHC and society.

*“Sometimes it happens that you show a video and then you say it anyway*,* so then it becomes that they* (the women) *kind of get information twice… You can replay it* (the video) *all over again… It is a good thing*,* but I think a lot of people might have lacked to get a little further*,* expand the information a bit and thus be able to get more information than just a picture then… but it is only positive… and then we still have interpreter conversations… and then that there is the opportunity to ask questions*,* that you can still ask questions to interpreter… but it gets easier.”* (Midwife 2).

The midwives revealed that dialog support was easily accessible because they had free access via computers when needed. On the other hand, when the technical equipment did not work optimally, it became time-consuming and contributed to suboptimal communication.

*“… I had to turn the computer where the direction of the sound is… and push it closest to the patient and it has worked then eh… some have heard truly well*,* some did not*,* they do not hear so well… hm… you truly have to concentrate and then you have to sit quietly*,* I cannot do anything else in the meantime*,* the patient has to truly listen…”* (Midwife 9).

### Women and their partners - the ability to be empowered

Communication via dialog support provided a sense of security considering that the information was perceived to be reliable. The midwives expressed further that conversations about complex pregnancy-related topics could be facilitated when providing information via dialog support. The midwives could not assess the quality of the translation performed by professional or informal interpreters. The information component was considered to lead to clear and confident communication and thus reduce the risk of misunderstandings.

*“… When we cannot talk to each other*,* we still have the interpreter who is something….well*,* not obstacles*,* you should not say that*,* but it will be anyway….they help to translate and it works very good in most cases*,* but….so I think that it (*the dialog support) *is truly an aid*,* because it is the correct words and someone is explaining in the right way… sometimes you do not feel that the interpreter might explain it in the right way*,* as you mean… I feel more confident with it* (the dialog support).*”* (Midwife 10).

One important component for the midwives was the opportunity to provide equal care. Migrant women generally receive less information than Swedish-born women do because of language barriers and time constraint. By using dialog support, the midwives felt that ASW received more information and enabled opportunities for conversation. Furthermore, the midwives perceived that giving ASW the same information as Swedish-speaking women could result in more women-centered health care.

*“… Because we would have had so much to gain from it*,* we are different as people*,* what we think is important and not*,* but I think that just like society*,* health care*,* everyone would have had a lot to gain from us seeing each woman for what they are and truly giving this equal care*,* to give every woman this right to get the information that we give to everyone…”* (Midwife 4).

The midwives perceived that communication via dialog support fulfilled the need for credible communication with ASW. Some midwives questioned the credibility of other translation apps, and they also experienced a limitation in brochures in certain subjects in Arabic. Using dialog support reduced the use of information brochures and digital translation apps. In addition, some midwives expressed a need for extended information and languages, even in this form of dialog support.

*“…Sometimes I think you have handed…a piece of paper and they go home and yes… but I* (the woman) *do not know what I’m going to read about*,* it just says something here*,* here it says cervical smear*,* yes*,* what is it…there we have many women who come for a cervical smear and think they’re going to do something completely different… but over the years I have had teenage boys come with their mothers because she had received a letter… and then he’s going to translate it*,* that is…ah*,* that is not possible… yes*,* it feels so damn unnecessary every now and then…then it is better*,* but then there’s this* (the dialog support), *then they know well*,* because many times when they determine*,* oh*,* maybe they would not have wanted that…it is much more fun when you can receive information in a simple way.”* (Midwife 4).

The midwives noted a significant increase in confidence and self-control when the women could communicate independently via dialog support without interpreter assistance. Some of the midwives illustrated the advantage of women being independent of interpreters. The midwives described that women generally felt uncomfortable discussing sensitive subjects, especially in the presence of a male interpreter.

*“…. I think that a lot of people who use interpreters…. They feel relied on this interpreter and I think they feel a little…. I have to have an interpreter*,* I am not so good*,* I am not good at Swedish… It is the only thing I can use*,* and it is probably a feeling that can be quite hard to always feel and then if you can get information like this* (by the dialog support) *and that you do not need to have that interpreter*,* I can go on a visit and I can at least get this information*,* I think they feel a little strengthened by it… Hm*,* maybe a little proud*,* or that it feels a bit… safer that way.”* (Midwife 4).

Some midwives perceived that communication via dialog support led to satisfaction in interactions with women and better relationships, which resulted in women feeling trust and safe in ANC.

*“… then I had a woman who wanted to….remove her intrauterine device… and then we had talked anyway*,* it worked pretty well with Swedish… but just when she had her period last time*,* she did not truly understand that and so she said no*,* I do not have my period now… and then I could use the question “Have you had your period in the last twelve months?”… and then I played it in Arabic and then I tried to say the sentence myself… (laughter)…. I truly tried*,* and she laughed… maybe it affected the communication a little bit*,* that in some way it strengthened the relationship*,* or the trust or something*,* that we…. well*,* almost spoke the same language.”* (Midwife 7).

## Discussion

This study is the first to explore midwives’ perceptions of communication via Swedish-Arabic dialog support at ANC. The midwives perceived that using dialog support facilitated communication as it provided a multimodal way of communication, and the content was culturally sensitive, and evidence based. A disadvantage was that the implementation of dialog support was perceived to be time-consuming; however, it was perceived as time-effective when the midwives gained digital skills. Midwives also perceived that communication via dialog support increased women’s empowerment ability.

A positive finding in this study was that dialog support was created in a multimodal way that could help communication with women with lower health literacy and lower language proficiency in Swedish. A study in Swedish pediatric care reported that the use of images and videos was perceived as beneficial for complementing or substituting verbal messages while discussing complex and culturally sensitive issues with migrant mothers [37]. Additionally, our findings confirm those of a previous study [26] on a translated version of the Swedish app HealthyMoms for promoting health behaviors among Arabic- and Somali-speaking women. The study revealed that health information needs to be presented in different media types (text, video, and audio) to facilitate communication [26]. Thus, combining formats is the most effective way of increasing levels of health literacy [38], since migrant women have been found to have lower health literacy than native-born pregnant women do [13, 27, 29, 39], particularly among first-generation migrants with low education levels [40]. Therefore, this study confirms that Sadima, as a multimodally designed form of dialog support, can be a useful tool to support communication difficulties in encounters with migrant women and thus to provide high-quality reproductive health care.

Another positive finding from this study was that the content of the dialog support followed evidence-based obstetric welfare guidelines. This is important since healthcare professionals have described that they prefer to use pregnancy apps relevant to their local healthcare context and come from a trusted source [25]. There is an extensive use of mobile translations for managing language barriers in healthcare, which is positively accepted [37]. However, automatic machine translations are known to be false and inaccurate, especially for medical vocabulary and less common languages [41]. The credibility of such translation tools was questioned by midwives in this study. They perceived that the dialog support fulfilled the need for credible communication with ASW and reduced the need for other digital translation apps. Another weakness in mobile translations is the lack of time efficiency because some translate one word or sentence at a time [42]. There is no conclusive evidence of what is effective when using artificial intelligence, websites, and other information technology for translating information to the migrant population. A systematic review revealed that the efficacy of mobile apps is evaluated more often for health promotion apps than medical translation apps [43]. Therefore, ANC needs evidence-based communication tools to ensure that quality communication occurs in interactions with migrant women and to minimize the degree of conversation uncertainty that leads to improved healthcare and reproductive outcomes [44, 45].

In this study, communication via dialog support was time effective. However, challenges in implementation were initially identified as time-consuming despite midwives reporting digital skills due to stressful working conditions. Swedish ANC is provided individually and continuity of care throughout pregnancy is applied. However, the length of the visits is standardized, usually 20–30 min [46], which is reported as a short time [10, 29, 40]. A limitation of dialog support was identified in terms of flexibility and need for technical support in clinical settings. Midwives reflected on the limitation in flexibility since the information was standardized and midwives usually adjusted information to women depending on their conditions and previous obstetric anamnesis. However, dialog support was constructed as a complement to current work routines and as complement to interpreters, which also is the case with other interventions such as HealthyMoms app [26] and the MAMAACT intervention [16].

Regarding the challenge in the implementation of dialog support, a similar result was found in other intervention studies to overcome language barriers in MHC [26–28, 47]. The challenge in implementation could be explained as the challenge of using eHealth, which might be related to digital abilities rather than motivation, trust, and access to technology [48]. Another challenge could be related to a cultural problem encountered by midwives which is related to punctuality. Midwives have been described that it was more common that migrant women arrive late to their appointments [49] which could contribute to time constraints for using dialog support. It might also be less motivating for midwives to implement dialog support since their encounter with ASW was not in a daily basis. Compared to other communication tools, Google Translate was reported to be the most common translation app used in healthcare [37]. Satisfaction and habit of using google translation among midwives could lead to resistance to use other dialog support tool, despite the fact of the inaccuracies in automatic translations [50]. Moreover, resistance to change in healthcare could be a reason for challenging implementation of dialog support. Previous study showed that a small number of healthcare professionals are willing to participate in changes [51]. Although, they are supposed to exchange something they know for something unknown [52]. Some ways to increase implementation of Health communication is to facilitate improvements in public health [53]. Therefore, it seems necessary to review priorities concerning communication with migrant women. One of the policies of the WHO for improving the healthcare of childbearing migrant women is to implement plain-language and sociocultural health information, including signs of pregnancy complications and navigation of the MHC system [54]. This study finding highlights that organizational support including easily accessible technical support seems needed to enable resources for midwives to enhance the use of digital dialog support.

This study revealed that using dialog support could increase ASW empowerment and improve relationships, resulting in feelings of trust and safety in ANC. The results of the present study align with those of earlier language-supported intervention studies [28, 55], which demonstrated that interventions such as parental education together with an interpreter [21] and community-based bilingual doulas [28] fostered trust and safety in MHC, empowered women, and reduced anxiety. The empowerment of women and the achievement of their individual needs for sexual and reproductive health seem essential for sustainable economic, social, and environmental development [56]. Therefore, using dialog support was beneficial in forming the conditions for ASWs seeking ANC services, while also upholding their autonomy and refraining from passing judgment on their choices. Integrating theoretical and practical knowledge and considering the connections and interactions between individual factors also empowers midwives to deliver tailored healthcare interventions.

Additionally, this study revealed that equally and culturally adapted information could result in more women-centered health care, which emphasizes emotional and social support in addition to medical care [54]. Another Swedish study revealed that currently available health information in MHC is not tailored for illiterate migrant women, which is challenging for midwives [26]. In addition to achieving health equity, health communication strategies improve people’s health outcomes and healthcare quality [26, 44]. Some ways to achieve that are by supporting informed choices, providing self-management tools, and increasing health literacy skills [44]. The standardized information in dialog support was, however, perceived as reducing midwives’ ability to tailor information to individual women and specific circumstances. An evaluation of language translation apps indicated that preset health phrases are suitable for everyday communication in healthcare settings, but they are insufficient for conversation and are, therefore, unable to replace professional interpreters [57, 58]. A future improvement could be to further adjust the content individually, which might lead to a more women-centered approach. To overcome language barriers, good and equal health and welfare could be achieved, and the independence and participation of migrant women in society could be increased. Communicating differently using the opportunities offered by digitization might improve healthcare and reproductive outcomes [45].

### Limitations of the study

A limitation of this study was the difficulty in recruiting midwives for the research because of midwives’ stressful working conditions, which resulted in longer time for data collection. However, to facilitate the recruitment process, workshops were offered to all midwives to increase their knowledge of dialog support. Similar action was made in other intercultural health communication interventions during implementation [16, 19, 20]. Despite the fact that this dialog support was developed in cooperation with midwives in accordance with the national basic program of ANC, technical support seemed needed to enhance the implementation.

Another limitation of this study could be related to the sample size. There are restricted ways to perceive a particular phenomenon in phenomenography studies. By including midwives with different backgrounds, different ANC clinics, and variations in experiences of using dialog support, group variations were reached. Furthermore, the number of participants was determined through a continuous, phenomenographic data analysis performed in parallel with data collection. The data collection was finalized when the researchers assessed that rich and broad had been collected and no new data emerged [32] which determined the sample size.

A third limitation of this study could be related to the fact that researchers (DB and MO) have been involved in the development of dialog support. Therefore, the participants were informed that the study focused on communication via dialog support, not its functionality or content. Regardless of the above limitations, the data provided a coherent picture, although the studied population included midwives of different ages, workplaces, and experiences using dialog support. The findings can be transferred to other settings or groups with similar characteristics [32].

## Conclusions

By being less dependent on professional and informal interpreters, communication via dialog support was perceived to facilitate communication between midwives and ASW and to increase women’s empowerment. This communication model was time-effective, but the dialog support implementation was time-consuming. The multimodality presentation was considered an advantage by enabling communication with women with varying literacy levels. Within our increasingly heterogeneous societies, healthcare needs to provide support for communication via digital dialog support that is women-centered and culturally sensitive to avoid misunderstandings and delayed or incorrect treatment of migrant people.

## Electronic supplementary material

Below is the link to the electronic supplementary material.

Supplementary Material 1

## Data Availability

To protect the integrity, anonymity and confidentiality of the participants, the data is not publicly available. However, it can be available on reasonable request.

## Declarations

### Clinical trial

This study is not a clinical trial, thus a clinical trial number is not applicable.

## Abbreviations

ANC: Antenatal care
App: Application
ASW: Arabic-speaking women
gANC: group antenatal care
MHC: Maternal health care
Sadima: Swedish-Arabic Dialog support In Maternal health care Application
WHO: World health organization
